# Supplementary material for: Blue Phosphorescent Pt(II) Compound Based on Tetradentate Carbazole/2,3′-Bipyridine Ligand and Its Application in Organic Light-Emitting Diodes
Source: Molecules. 2024 Jun 20;29(12):2929. doi: 10.3390/molecules29122929 (PMC11206379; doi:10.3390/molecules29122929)
Supplement: Supplementary file 1 [file molecules-29-02929-s001.zip › molecules-3037340-supplementary.pdf]

Electronic Supporting Information

for

Blue phosphorescent Pt(II) compound based on tetradentate carbazole/2,3′-bipyridine ligand and its application in organic light-emitting diodes

Hakjo Kim[a], Chan Hee Ryu[a], Miso Hong[a], Kang Mun Lee[a], Seung Chan Kim[b], and Youngjin kang[a*]

[a]*Division of Science Education and Department of Chemistry, Kangwon National University, Chuncheon 24341, Republic of Korea;* [b]*School of Chemical Engineering, Sungkyunkwan University, 2066 Seobu-ro, Jangan-gu, Suwon, Gyeonggi 16419, Republic of Korea*

Corresponding Author: kangy@kangwon.ac.kr

Contents

- **Experimental**
- **Figure S1**. ^{1}H NMR of 2-((2',6'-dimethoxy-[2,3'-bipyridin]-6-yl)oxy)-9-(pyridin-2-yl)- 9H-carbazole (pypyOczpy) in $CDCl_3$.
- **Figure S2**. ^{13}C NMR of 2-((2',6'-dimethoxy-[2,3'-bipyridin]-6-yl)oxy)-9-(pyridin-2-yl)- 9H-carbazole (pypyOczpy) in $CDCl_3$.
- **Figure S3**. ^{1}H NMR of **1** in CD_2Cl_2.
- **Figure S4**. ^{13}C NMR of **1** in CD_2Cl_2.
- **Figure S5**. Simple crystal-packing patterns for **1** in a unit cell.
- **Figure S6**. The dihedral angle between two planes (bipyridine and pyridine ring of pyridyl-carbazole).
- **Figure S7**. The Pt-Pt distance between two adjacent molecules.
- **Figure S8**. TGA data of **1**.
- **Figure S9**. CV data of **1**.
- **Figure S10**. Emission spectra of **1** at different doping levels.
- **Figure S11**. Emission decay curve detected at 520 nm in film (50 wt%) of **1**
- **Table S1.** Crystallographic data and parameters for **1**.
- **Table S2.** Selected bond lengths (Å) and angles (°) for **1**.
- **Table S3.** Summary of TD-DFT calculation results.

Experimental

1. General information

All experiments were performed under dry N_2 atmosphere using standard Schlenk technique. All solvents were freshly distilled over appropriate drying reagents prior to use. All starting materials were purchased from either Aldrich or Alfa and used without further purification. The NMR spectra were recorded on JEOL 400 MHz spectrometer. UV/Vis and photoluminescent spectra for all samples with concentrations of 10 μM were obtained from the UV/Vis spectrometer Shimadzu UV 1601-PC and a Shimadzu RF-6000-Spectrofluorophotometer, respectively. All solutions for photophysical experiments were degassed with more than three repeated freeze-pump- thaw cycles in a vacuum line. Thin film was made by spincasting from a solution of 10% wt% both complexes in PMMA (polymethylmethacrylate) in dichloromethane.

2. X-ray analysis

X-ray diffraction data for **1** were collected at 173(2) K on a Bruker SMART APEX II ULTRA diffractometer equipped with a graphite monochromated Mo Kα (λ = 0.71073 Å) radiation generated by a rotating anode and a CCD detector. The cell parameters for the compound were obtained from a least-squares refinement of the spots (from 36 collected frames). Data collection, data reduction, and semi-empirical absorption correction (SADABS) were carried out using the software package of APEX2. All of the calculations for the structure determination were carried out using the APEX2 package with the SHELXS-2014 and SHELXL-2014 programs. The non-hydrogen atoms were refined anisotropically. All hydrogen atoms were placed in calculated positions and refined isotropically in a riding manner along with their respective parent atoms. CCDC 2354408 contains the supplementary

crystallographic data for this paper. These data can be obtained free of charge from The Cambridge Crystallographic Data Centre via www.ccdc.cam.ac.uk/data_request/cif.

3. Device fabrication and measurement

The **1** as emitter was evaluated by doping in the mixed host of mCBP/CNmCBP CN at 3-20% doping concentration using the device configuration of indium tin oxide (50 nm) / poly(3,4-ethylenedioxythiophene): poly(styrenesulfonate) (PEDOT:PSS, 40 nm) / 4,4′-cyclohexylidenebis [*N*,*N*-bis(4-methylphenyl)benzeneamine] (TAPC, 10 nm) / 1,3-bis(*N*-carbazolyl)benzene (mCP, 10 nm) / mCBP:CNmCBPCN-dopant (25 nm)/diphenyl[4-(triphenylsilyl)phenyl]phosphine oxide (TSPO1, 5 nm) / 1,3,5-tris(1-phenyl-1*H*-benzo[*d*]imidazol-2-yl)benzene (TPBi, 20 nm) / LiF (1.5 nm) / Al (200 nm). All devices were fabricated by vacuum thermal evaporation under 1×10^{-6} torr. The encapsulation with glass was performed under the N_2 atmosphere to protect the device from O_2. The device performances were measured using Keithley 2400 source meter and CS 2000. (Konica Minolta Inc.)

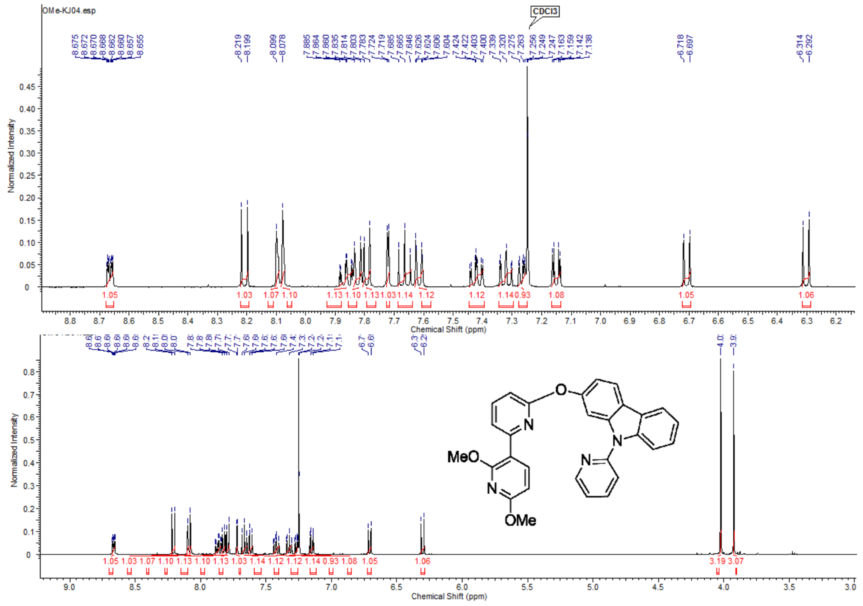

Figure S1. ^{1}H NMR of 2-((2',6'-dimethoxy-[2,3'-bipyridin]-6-yl)oxy)-9-(pyridin-2-yl)- 9H-carbazole (pypyOczpy) in $CDCl_3$.

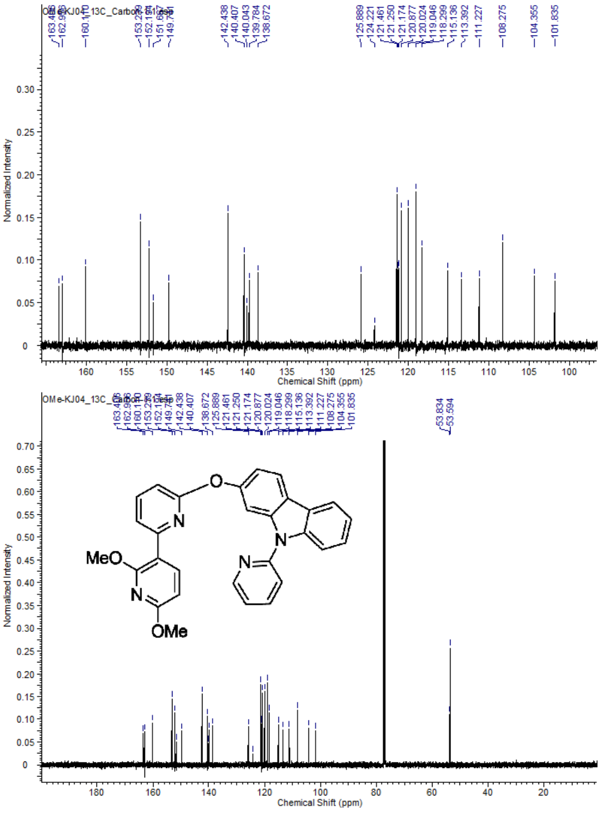

Figure S2. ^{13}C NMR of 2-((2',6'-dimethoxy-[2,3'-bipyridin]-6-yl)oxy)-9-(pyridin-2-yl)- 9H-carbazole (pypyOczpy) in $CDCl_3$.

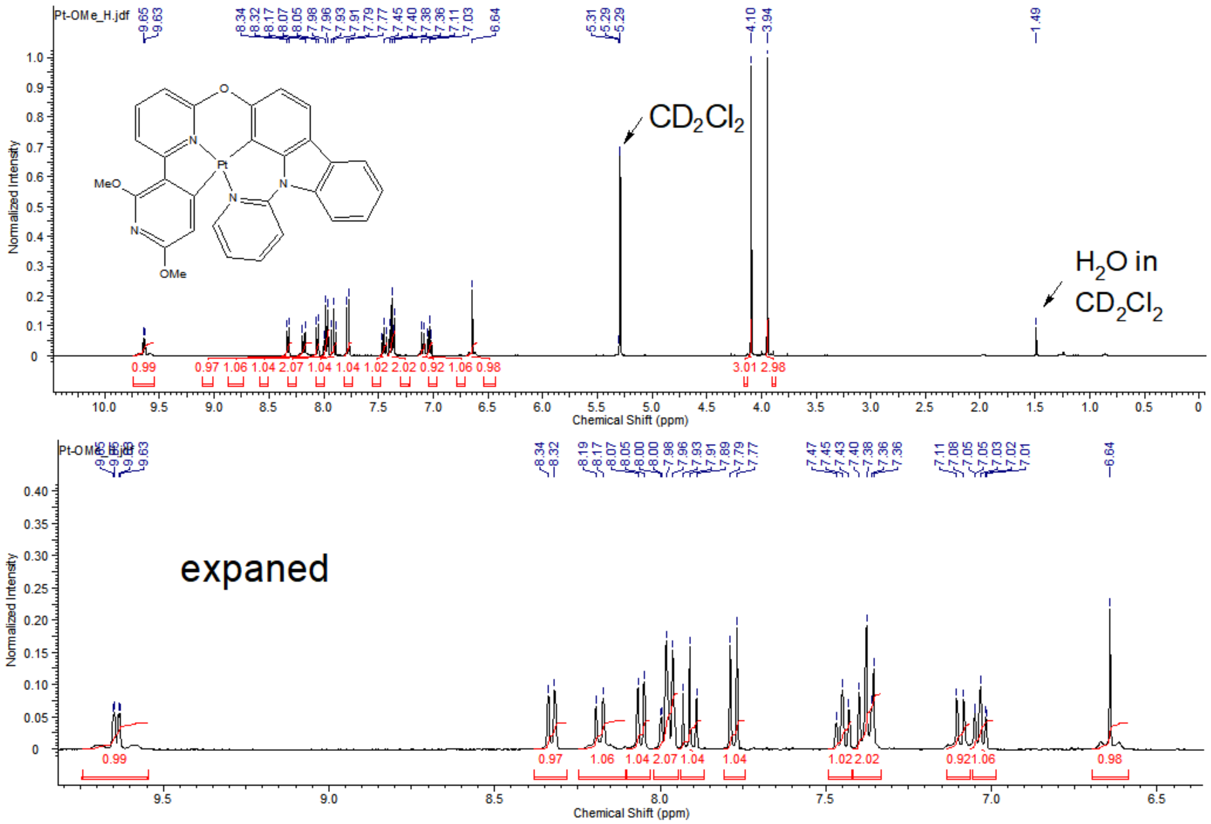

Figure S3. ^{1}H NMR of **1** in CD_2Cl_2.

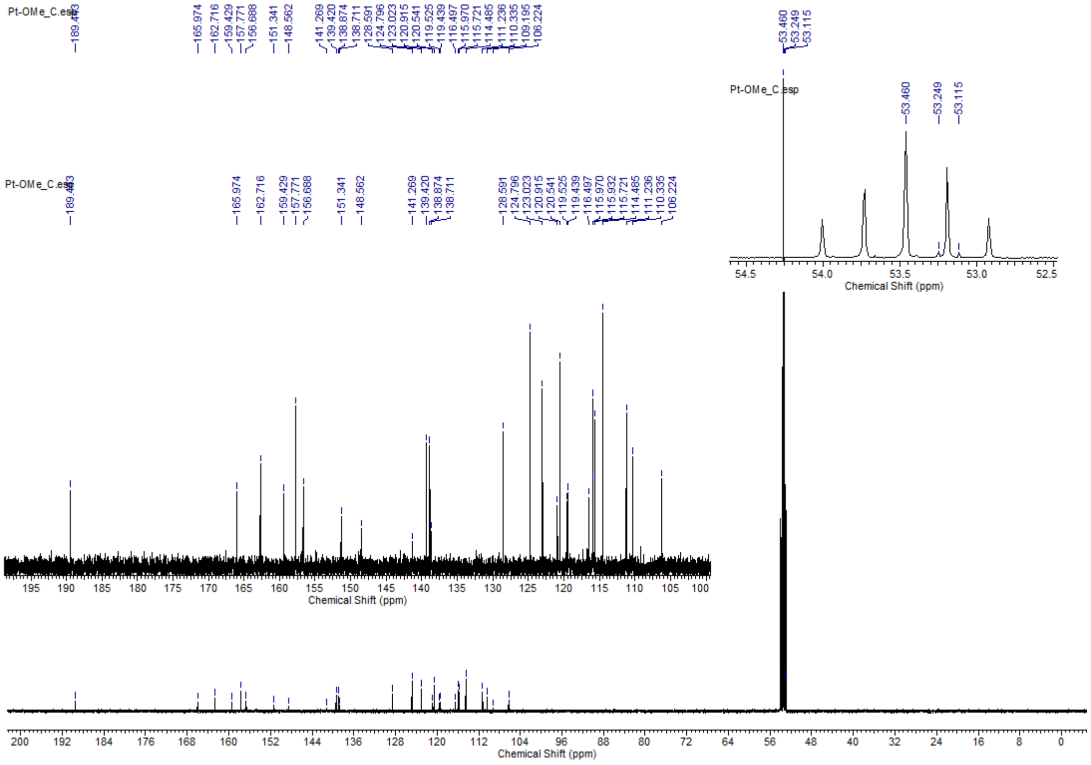

Figure S4. ^{13}C NMR of **1** in CD_2Cl_2.

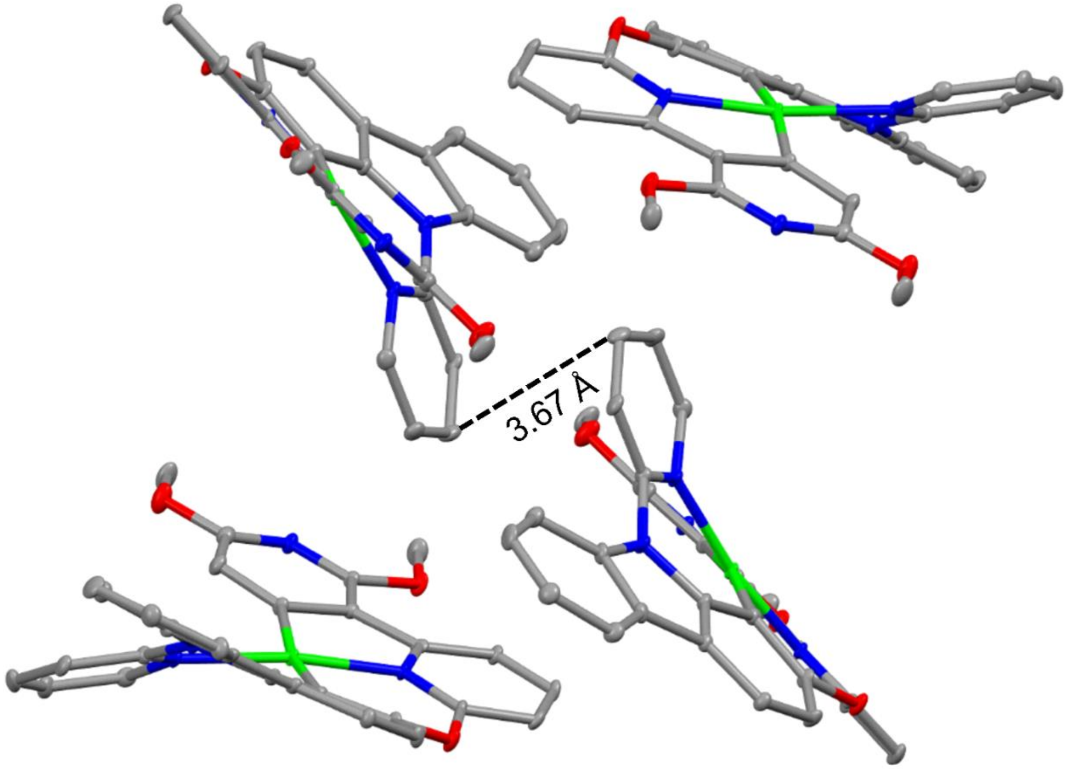

Figure S5. Simple crystal-packing patterns for **1** in a unit cell.

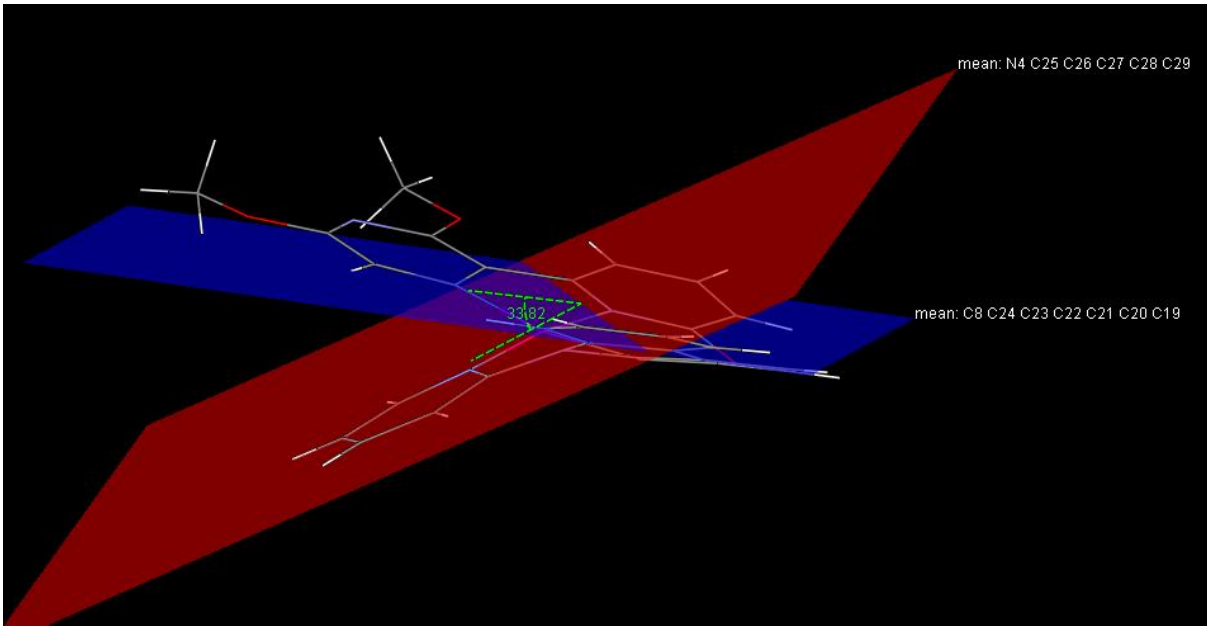

Figure S6. The dihedral angle between two planes (bipyridine and pyridine ring of pyridyl-carbazole).

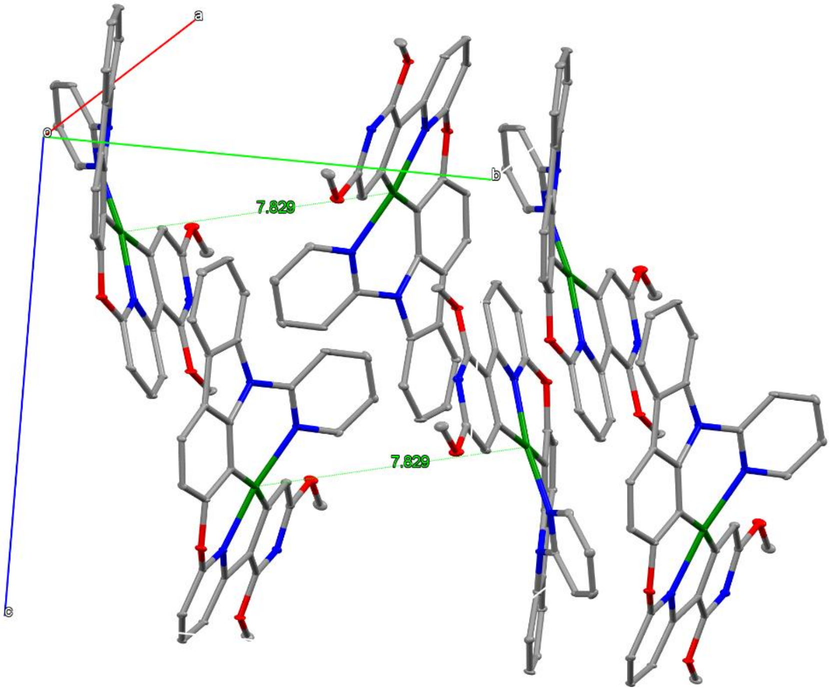

Figure S7. The Pt-Pt distance between two adjacent molecules.

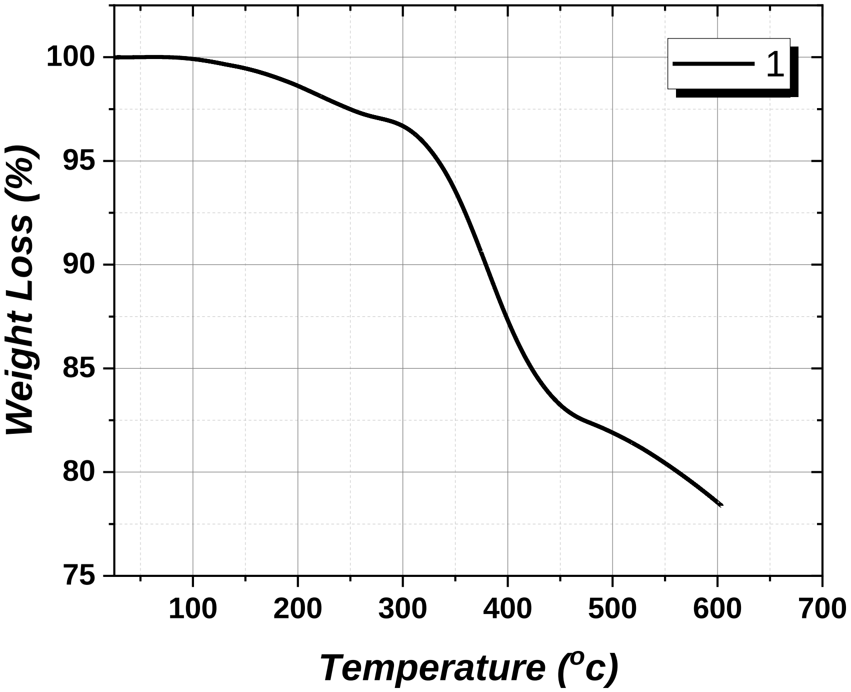

Figure S8. TGA data of **1**.

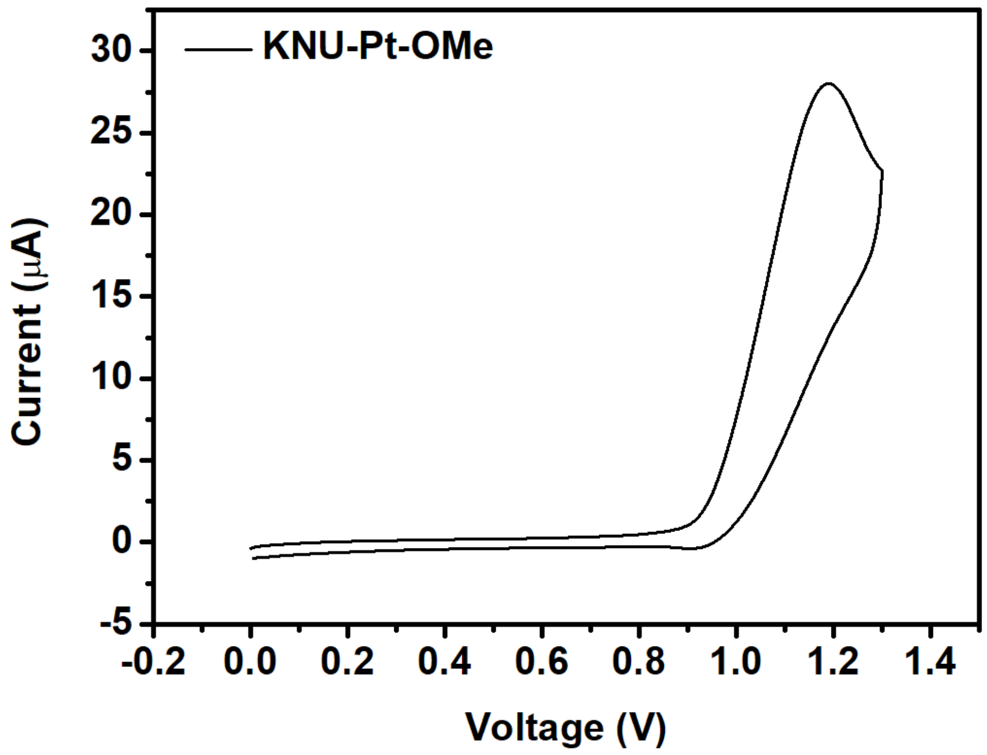

Figure S9. CV data of **1** (Oxidation).

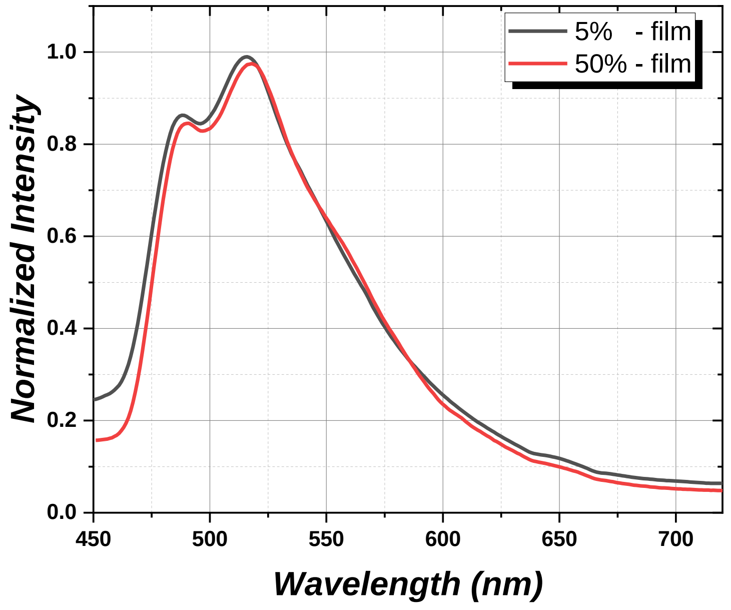

Figure S10. Emission spectra of **1** at different doping levels.

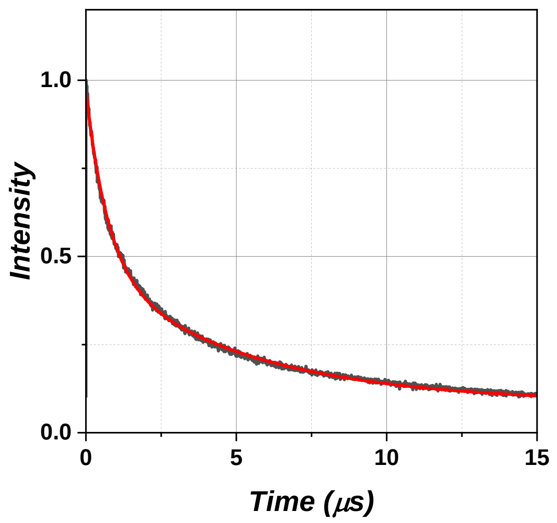

Figure S11. Emission decay curve detected at 520 nm in film (50 wt%) of **1**(black line).

Table S1. Crystallographic data and parameters for **1**.

Compound	**1**
Formula	$C_{29}H_{20}N_4O_3Pt$
Formula weight	667.58
Crystal system	Monoclinic
Space group	$P2_1/n$
a (Å)	12.7541(4)
b (Å)	13.3351(4)
c (Å)	13.4984(4)
α (°)	90
β (°)	104.0550(10)
γ (°)	90
V (Å^3)	2227.04(12)
Z	4
ρ_{calc}(g cm^{-3})	1.991
μ (mm^{-1})	6.344
F(000)	1296
T (K)	123(2)
Scan mode	*φ and ω-scan*
hkl range	$-16 < h < 16$, $-13 < k < 17$, $-17 < l < 17$
Measd reflns	20927
Unique reflns [R_{int}]	5117 [0.0475]
Reflns used for refinement	5117

Refined parameters	336
$R_1{}^a$ (I > 2σ(I))	0.0251
$wR_2{}^b$ all data	0.0837
GOF on F^2	1.003
ρ_{fin} (max/min) (e Å^{-3})	1.476, −2.333

$^a R_1 = \sum||F_o| - |F_c||/\sum|F_o|$. $^b wR_2 = \{[\sum w(F_o{}^2 - F_c{}^2)^2]/[\sum w(F_o{}^2)^2]\}^{1/2}$.

Table S2. Selected bond lengths (Å) and angles (°) for **1**.

Compound	**1**
length (Å)	
Pt1–C1	2.048(4)
Pt1–C14	2.004(4)
Pt1–N2	2.001(3)
Pt1–N4	2.044(3)
O3–C12	1.338(4)
O3–C13	1.395(4)
angles (°)	
C1–Pt1–N2	81.38(13)
C1–Pt1–N4	98.91(13)
C14–Pt1–N2	92.34(14)
C14–Pt1–N4	88.49(14)
C3–O1–C6	116.7(3)
C4–O2–C7	117.5(3)
C12–O3–C13	127.4(3)

Table S3. Summary of TD-DFT calculation results

	**eV**	**nm**	**f**	**MO**	**CI**	**%**	**mlct(%)**
$S_0 \rightarrow S_1$	2.9958	413.86	0.033	HOMO → LUMO	0.5676	68.0	14.7
				HOMO -1 → LUMO	0.3872	32.0	
$S_0 \rightarrow T_1$	2.6938	460.26		HOMO → LUMO	0.3650	25.0	14.9
				HOMO → LUMO+1	0.3600	24.0	
				HOMO-1 → LUMO+1	0.3780	27.0	
